# Quality of life, pain and anxiety in patients with nephrostomy tubes

**DOI:** 10.1590/1518-8345.3039.3191

**Published:** 2019-10-07

**Authors:** Luis Manuel Fernández-Cacho, Rosa Ayesa-Arriola

**Affiliations:** 1 Hospital Marqués de Valdecilla University, Department of Radiology Santander, Cantabria, Spain.; 2 Hospital Marqués de Valdecilla University, Department of Psychiatry, Santander,Cantabria, Spain.; 3 Instituto de Investigación Valdecilla (IDIVAL), Santander, Cantabria, Spain.; 4 University Of Cantabria, School of Medicine, Santander, Cantabria, Spain.; 5 Centro de Investigación Biomédica en Red Salud Mental (CIBERSAM), Spain.

**Keywords:** Percutaneous Nephrostomy, Ostomy, Quality of Life, Anxiety, Pain, Education Nursing, Nefrostomia Percutânea, Ostomía, Qualidade de Vida, Ansiedade, Dor, Educação em Enfermagem, Nefrostomía Percutánea, Ostomía, Calidad de Vida, Ansiedad, Dolor, Educación en Enfermería

## Abstract

**Objective:**

to evaluate the impact on the quality of life as well as anxiety and pain in patients with nephrostomy tubes.

**Method:**

this is a longitudinal descriptive study performed on a sample of n=150 patients. To evaluate the quality of life, the EuroQol-5D questionnaire was used; anxiety was quantified by the Beck Anxiety Inventory; to study pain, a visual analogue scale was employed.

**Results:**

statistically significant differences were found in the quality of life, with its worsening (r = 0.51; p <0.01) when evaluated at the first tube replacement. Patients presented mild to moderate anxiety before the procedure, which was reduced at the first tube replacement, although this difference was not significant (r = 0.028, p = 0.393). Finally, the degree of pain was also significantly reduced (r = 0.13, p<0.01) after six weeks. As for gender, women presented the worst values ​​in the three variables studied (worse quality of life and greater anxiety and pain).

**Conclusions:**

nephrostomy tubes have a negative impact on the patient’s quality of life. During the time they live with these tubes, patients have mild to moderate pain and anxiety.

## Abstract (pt)

**Objetivo:**

avaliar o impacto na qualidade de vida, bem como a ansiedade e dor em pacientes com sondas de nefrostomia.

**Método:**

estudo descritivo longitudinal realizado em uma amostra de n=150 pacientes. Para avaliar a qualidade de vida, utilizou-se o questionário EuroQol-5D; a ansiedade foi quantificada pelo Inventário de Ansiedade de Beck; para estudar a dor, foi utilizada uma escala visual analógica.

**Resultados:**

foram encontradas diferenças estatisticamente significativas na qualidade de vida, com sua piora (r = 0,51; p <0,01) quando avaliada na primeira troca da sonda. Os pacientes apresentaram ansiedade leve a moderada antes do procedimento, que foi reduzida na primeira troca da sonda, embora esta diferença não tenha sido significativa (r = 0,028; p = 0,393). Finalmente, o grau de dor também foi significativamente reduzido (r = 0,13; p<0,01) após seis semanas. Quanto ao sexo, as mulheres apresentaram os piores valores nas três variáveis ​​estudadas (pior qualidade de vida e maior ansiedade e dor).

**Conclusões:**

Sondas de nefrostomia têm um impacto negativo na qualidade de vida do paciente. Durante o tempo que convivem com estas sondas, os pacientes têm dor e ansiedade leve a moderada.

## Abstract (es)

**Objetivo:**

valorar el impacto en la calidad de vida, así como la ansiedad y el dolor que presentan los pacientes portadores de sondas de nefrostomía.

**Método:**

estudio descriptivo longitudinal que se llevó a cabo sobre una muestra de n=150 pacientes. Para valorar la calidad de vida se empleó el cuestionario EuroQol-5D; la ansiedad fue cuantificada mediante el Inventario de Ansiedad de Beck; para estudiar el dolor se empleó una escala visual analógica.

**Resultados:**

encontramos diferencias estadísticamente significativas en la calidad de vida, produciéndose su empeoramiento (r=0.51; p<0.01) cuando fue valorada en el primer cambio de sonda. Los pacientes presentaron una ansiedad leve a moderada previa al procedimiento, que se vio reducida en el primer cambio de sonda, si bien esta diferencia no resultó significativa (r=0.028; p=0.393). Por último, el grado de dolor también se vio disminuido de forma significativa (r=0.13; p<0.01) al cabo de seis semanas. Por sexos, las mujeres presentaron peores valores en las tres variables estudiadas (peor calidad de vida, y mayor ansiedad y dolor).

**Conclusiones:**

las sondas de nefrostomía suponen un impacto negativo en la calidad de vida del paciente. Durante el tiempo que conviven con dichas sondas, los pacientes presentan dolor y ansiedad leve a moderada.

## Introduction

Percutaneous nephrostomy is a technique that involves placing a flexible tube that directly communicates the kidney with the outside through a hole in the skin, guided by ultrasound. The first case was described in 1955^(^ 1 ^)^, so it is a very recent technique. It is a widely established procedure for patients with urological supravesical obstruction, urinary diversion and urinary fistula, besides other indications. Most are obstruction cases, which prevent the correct urine pathway from the upper urinary tract to the lower part, avoiding, thus, the accumulation of urine in the kidney, with a consequent risk of hydronephrosis and kidney impairment^(^ 2 ^)^. Percutaneous nephrostomy is performed by ultrasound-guided direct puncture and subsequent radioscopic control, usually in the prone position and under local anesthesia at the puncture site. The result is the placement of a pig tail tube that communicates the kidney with the exterior^(^ 3 ^)^. Due to the low incidence of complications (between 4% and 10%), it is a very appropriate technique as a urinary diversion^(^ 3 ^)^. The tube, at its distal end, is attached to a urine collecting bag that is usually attached to the patient’s leg. To avoid possible obstruction of the tube by the deposition of metabolic waste, patients should go weekly to the health centers to perform drainage and control of the peri-catheter area^(^ 4 ^-^ 7 ^)^. In addition, with almost monthly frequency, the tubes should be replaced at the radiology services. The ease of the procedure and its low morbidity makes it an increasingly widespread and accepted technique. The number of patients submitted to this technique is increasing exponentially. In addition, due to the various pathologies that may require the placement of the tubes, the target population varies a wide range of ages, from newborns to elderly patients. Its duration can be from a few weeks to many years, or they may even carry the tubes permanently. Therefore, patients should learn to live with tubes for a certain time^(^ 8 ^)^.

The term health is a multidimensional concept that the World Health Organization (WHO) describes as a state of complete physical, mental, and social well-being and not just the absence of disease or infirmity. In the definition of health, different spheres coexist, such as culture, society, economy or the dominant politics of each country or continent where the term is evaluated.

Health-related quality of life (HRQOL) is a concept that, although its definition is also multidimensional and can be described as a person’s subjective assessment of his/her physical and mental health, turns out to be a concept of an individual nature, which includes both positive and negative dimensions. The definition of HRQOL, provided by the WHO, presupposes the individual’s perception of the effects of a disease and its consequences, and how it affects different areas of life, especially the consequences it has on physical, emotional, and social domains. By assessing HRQOL, the individual also performs an assessment of his/her position in life in the context of the culture and value systems in which he/she lives and in relation to his/her goals, expectations, standards and concerns, as described by WHO in 1994. Measuring quality of life is increasingly important as it is a way of assessing the health of a given population^(^ 9 ^)^. Moreover, it allows to detect problems and analyze the effectiveness and efficacy of health interventions. The instruments for measuring HRQOL are essential in the different stages of the nursing care process, despite the limitations they present. One of the main obstacles that can arise when administering a given questionnaire is the sociocultural context in which it will be carried out. Habits, customs, traditions or beliefs can condition certain items, as well as the way to ask about them. In order to measure HRQOL in these conditions, very robust questionnaires are required, which can be administered quickly and easily and allow reliable and valid results to be obtained. It is important to use measuring instruments that have been validated and adapted for the population to be studied^(^ 10 ^-^ 12 ^)^.

HRQOL refers to a concept widely studied in several pathologies or diseases, such as pulmonary diseases^(^ 13 ^)^, chronic kidney failure^(^ 14 ^-^ 19 ^)^, heart failure^(^ 20 ^)^ or in patients with Crohn’s disease who undergo intestinal resection surgery^(^ 21 ^-^ 22 ^)^, among others. This instrument is essential because it incorporates the patient’s perception as one of the obligatory and necessary parameters in the different steps that constitute the process of any health intervention. Likewise, it represents a dimension in which all health education must address. It should be present both in the assessment of needs, diagnosing problems, planning of interventions, execution of activities or tasks and, finally, in the evaluation of health outcomes. It is necessary to have valid and reliable instruments for this measurement to provide scientifically based empirical evidence for the health decision-making process.

The main objective of this study was to evaluate the impact on quality of life, as well as to analyze the level of anxiety and pain before and after the nephrostomy tubes implanting procedure.

Two hypotheses were proposed: 1) The quality of life of patients with a nephrostomy tube will be lower in the first tube replacement than before their implantation. 2) Patients will also have, then, a higher level of anxiety and pain, compared to their self-assessment before the procedure.

## Method

This is a longitudinal descriptive study, approved by the Ethics and Clinical Research Committee of Cantabria, Spain (2015.099). It was performed in a sample of n=150 patients selected from the Radiology Department of the *Hospital Universitário Marques de Valdecilla* (HUMV) in Santander (Cantabria), Spain. Non-probabilistic sampling was performed, including patients seen at the HUMV Radiology Service scheduled to have a nephrostomy tube inserted, coded according to the catalog of the Spanish Society of Medical Radiology (SERAM) number 07030300 and who attended the first replacement of the tube (six weeks after implantation), where the second evaluation was performed. The calculation of the number of subjects needed to carry out this project was made using the Gpower software, version 3.1.9.2. For this purpose, the sample size was estimated for a normal distribution (Pearson) based on a mean difference^(^ 23 ^-^ 24 ^)^, with a 95% confidence interval (CI), a 5% error margin and a size effect of 0.3. With these parameters, the calculation indicated that the required sample size was 111 patients. To obtain as much information as possible and anticipate the loss of subjects during follow-up, a sample of 150 patients was established. The study was conducted between January 2016 and April 2018, according to the following inclusion criteria: patients of age (age ≥ 18 years) who should have received a nephrostomy tube and presented themselves for the first tube replacement. Patients would have to voluntarily participate in the research after receiving all information about the study from the main researcher, both verbally and in writing, and would have signed their informed consent form. In addition, the HUMV medical and nursing management personnel, as well as the head and the supervisor of the hospital’s Radiology Diagnostic service were informed about the study. As exclusion criteria, it was determined that patients who had previously had another nephrostomy tube or any other type of ostomy and/or those patients whose cognitive status would prevent them to produce reliable answers would not have participated in the study. Another exclusion criterion was removal of the tube before the first replacement or the need to remove it urgently, before the predetermined date for the first replacement (six weeks post-implantation). All patients were informed on the possibility to revoke their participation in the study at any time.

In this study, we started from the patient’s situation before surgery and then compared it approximately six weeks after the tube was implanted, when the patient went to the radiology service for the first time to undergo the first tube replacement. Since all nephrostomies were performed in this service, either by the Interventional Vascular Radiology team or by the Central Radiology team, we believe that both research data collection, pre and post-procedure (first tube replacement) would have been performed in the same Radiology service to avoid biases due to different information received in other services, such as in the emergency room or in the urological hospitalization unit.

As variables, the quality of life, anxiety and pain before and after the procedure were studied, as well as the use of psychopharmaceutical drugs. In addition, other socio-demographic variables were included to assess how they influence these changes and to assess whether there are particularly vulnerable populations for whom intervention is particularly essential in order to minimize the negative impact of living with nephrostomy tubes. The following variables were analyzed: age, gender, marital status (single, married, separated, and widowed), family unit (number of people living with the patient, included him/herself), education (no studies, primary studies, high school, university studies), leisure activity (no leisure activities, leisure time less than twice a week, 2 to 5 times a week, and more than 5 times a week) and work situation (employed, medical leave, unemployed, and retired).

The instruments used to quantify these variables were: Quality of life EuroQ-5D questionnaire (the mean of each of the five dimensions was used as a measure), the Beck Anxiety Inventory (BAI) for anxiety, and the visual analogue scale to assess pain.

The European Quality of Life-5 Dimensions (EQ-5D) questionnaire is a widely accepted international instrument for assessing health-related quality of life. In addition, it has been validated for different cultural contexts^(^ 25 ^)^, including the Spanish one, and is very useful as an instrument for measuring health status within a population^(^ 26 ^)^. It is a questionnaire designed to be administered in a variety of measurement conditions: by mail, self-administered or by interview. The EQ-5D is divided into three parts: the first part allows the respondent to define their health status in five dimensions (mobility, personal care, daily activities, pain/discomfort and anxiety/depression), each one scoring three levels of severity oscillate between score 1 (no problems), 2 (some problems) and 3 (many problems). Higher scores are related to a worse perception of quality of life. For example, the questionnaire of an individual with no mobility problems, personal care or daily activities, but with moderate pain and anxiety, would be summarized as 11122. The second part is a visual analog scale graded from 0 (worst possible health) to 100 (best possible state of health), which allows the individual to assess his/her current health status. In order for patients to assess in a more tangible and understandable way the impact of the nephrostomy tube on their quality of life and to be able to detect differences in scores that mean clinically relevant changes, we considered it appropriate to simplify the values ​​of this scale and to employ one that ranges from 0 to 10, taking into account, on the other hand, that we also use a visual pain scale with the same numerical range, as we will explain later on, trying to avoid possible misinterpretation and confusion. The third part of the questionnaire collects other data in the form of variables that allow the demographic characterization of the individuals evaluated^(^ 27 ^-^ 28 ^)^.

Beck’s anxiety test is a useful tool that evaluates the most frequent symptoms of anxiety. Moreover, since 2011, it has been adapted to the Spanish population^(^ 29 ^)^. The questionnaire consists of 21 questions, providing a score ranging from 0 to 63 (each item scores from 0 to 3 based on the greater or lesser severity of the symptoms). Cut-off points for stratifying the level of anxiety are as follows: 0-21 (low anxiety), 22-35 (moderate anxiety), and 36 or greater (severe anxiety). The total score is the sum of all items, evaluating the symptoms present in the last week and in the current moment ^(^ 29 ^)^. Both the EQ-5D questionnaire and the Beck Anxiety Inventory have proved to be sufficiently reliable, easy to administer, and proven to validate both quality of life and anxiety, respectively. To measure pain, a visual analogue scale was used, in which 0 meant no pain and 10 the greatest pain borne by the patient. The latter variable was introduced to avoid the bias of pain as an improvement in quality of life.

For statistical analysis, quality of life was considered globally, as well as each of the five dimensions of the EQ-5D questionnaire, based on the various sociodemographic variables used in the study. Data were analyzed in the Statistical Package for Social Science, version 19.0 (SPSS Inc., Chicago, IL, USA). The chi-square test (Χ^2^) was used to evaluate categorical variables, as well as the Student’s T-test for related samples, which was used to compare quality of life, anxiety and pain before and after the procedure. Cohen’s d and r (effect size) were used to evaluate the effect size. The degree of correlation was determined by taking into account the following values: perfect (when the value is close to +/- 1, whereby if one variable increases, the other also increases if it is positive, or decreases if negative), high correlation (when the coefficient value is between +/- 1 and +/- 0.5 and it can be said that the correlation is strong), moderate degree (when the value oscillates between +/- 0.3 and +/- 0.49 and the correlation is medium), low correlation (when the value is less than +/- 0.29 and the correlation between the variables is small) and no correlation (when the value is zero). The results were considered significant for values ​​of p<0.05.

## Results

Of the total sample analyzed (n = 150), 68% (n = 102) were men and 32% (n = 48) women. Two patients were considered lost since they did not continue in the study due to death before the first tube replacement. The mean age of the patients was 61.67 years old, significantly higher in men than in women (62.62 ± 13.8 vs 59.67 ± 14.86). A fraction of 67.5% (n=100) of the total sample was married and about 43.7% (n = 65) lived at home with another relative, i.e., at least two family members in the household. Regarding the level of studies, 37.1% (n = 56) attended high school, including baccalaureate and/or vocational training, 28.5% (n=42) secondary studies and 15.9% (n=24) had completed university studies, resulting in a significantly higher education level in women than in men (20.8% vs. 13.7%). Observing their routine of daily activities, no differences were found by gender, and 33.3% (n = 50) reported performing daily exercises. It should be noted that patients who were employed before receiving the nephrostomy tube (n = 62), 93% (n = 58) were on temporary medical leave at the time of the first tube replacement. Finally, regarding psychopharmaceuticals, 30% (n = 45) of the patients consumed systematically psychoactive drugs (anxiolytics and/or antidepressants), with a higher percentage in men (31.4%; n =32) than in women (27.1%, n = 13). The descriptive statistics of the sample is presented in Table 1.

**Table 1:** Socio-demographic characteristics of the sample studied. Santander, Spain, 2016-2018

	Total (n=150)		Men (n=102)		Women (n=48)	
	Mean	St. Dev*	Mean	St. Dev*	Mean	St. Dev*
Age (years old)	61.6	14.16	62.62	13.8	59.67	14.86
	N	%	N	%	N	%
Working status						
Employed	4	2.6	1	1.0	3	6.3
Medical leave	58	38.4	36	35.3	22	45.8
Unemployed	11	7.3	7	6.9	4	8.3
						
Marital status						
Single	19	12.6	15	14.7	4	8.3
Married	102	67.5	69	67.6	33	68.8
Separated	7	4.6	4	3.9	3	6.3
Widower	22	14.6	14	13.7	8	16.7
Family unit^†^						
1	16	10.6	11	10.8	5	10.4
2	66	43.7	48	47.1	18	37.5
3	39	25.8	29	28.4	10	20.8
4	23	15.2	13	12.7	10	20.8
5	6	4.0	1	1.0	5	10.4
Education						
No studies	27	17.9	15	14.7	12	25.0
First degree	56	37.1	42	41.2	14	29.2
High school	43	28.5	31	30.4	12	25.0
University degree	24	15.9	14	13.7	10	20.8
Leisure activities						
No leisure	29	19.2	18	17.5	11	22.9
< twice/week	32	21.2	24	23.5	8	16.7
2 - 5 times/week	39	25.8	26	25.5	13	27.1
> 5 times/week	50	33.1	34	33.3	16	33.3
Take psychopharmaceuticals^‡^						
Yes	43	30.0	32	31.4	13	27.1
No	105	70.0	70	68.6	35	72.9

* St. Dev = Standard Deviation;

† Family unit = number of members in the household, including the patient him/herself;

‡ Psychopharmaceuticals = take any drugs which affect psychological behavior (anxiety medication and/or antidepressants)

Analyzing quality of life in general, as shown in Table 2, it has decreased (7.51 ± 2.104 vs 5.07 ± 1.936) six weeks after the procedure (at first tube replacement), a statistically significant difference (Student’s T-test=17.84, p<0.01), showing a large effect size (d > 1). Similar results were found stratifying the variable by gender: the quality of life showed a reduction, which is also statistically significant in both men (7.73 ± 1.936 vs 5.24 ± 15.512; Ttest = 15.512; p>0.01); as in women (7.06 ± 2.301 vs. 4.73 ± 2.029, Ttest = 9.29, p>0.01) and with a large effect size (d > 1). It should be noted that the women studied had a lower quality of life than men, both before and after having the tube implanted (7.73 ± 1.936 vs 7.06 ± 2.301 pre-procedure and 5.24 ± 1.878 vs. 4.73 ± 2,029 post-procedure).

**Table 2:** Quality of life pre- and post-procedure. Santander, Spain, 2016-2018

	Pre-procedure		Post-procedure		P value^†^	d^‡^	r^§^
	Mean	St. Dev*	Mean	St. Dev.*			
Total (n=150)	7.51	2.104	5.07	1.936	P<0.01	1.20	0.51
Men (n=102)	7.73	1.936	5.24	1.878	P<0.01	1.30	0.54
Women (n=48)	7.06	2.301	4.73	2.029	P<0.01	1.03	0.45

* St. Dev = Standard Deviation;

† P value;

‡ d = Cohen's D;

§ r = Effect size

As it can be seen in Table 3, the patient’s anxiety prior to the procedure was greater than at the first tube replacement (6^th^ week), both in general (9.62 ± 7.15 vs, 9.19 ± 7.70, Ttest = 0.857, p = 0.393), as stratified by gender (8.84 ± 7.139 vs.8.79 ± 7.854, Ttest = 0.089, p = 0.929 in men and 11.27 ± 6.961 vs.10.02 ± 7.373; Ttest=1.176, p=0.245 in women). This difference, however, is not statistically significant (p > 0.05). It should be noted that women presented greater anxiety than men, both pre- and post-procedure.

**Table 3:** Anxiety pre and post-procedure. Santander, Spain, 2016-2018

	Pre-procedure		Post-procedure		P value^†^	d^‡^	r^§^
	Mean	St. Dev*	Mean	St. Dev*			
Total (n=150)	9.62	7.150	9.19	7.700	0.393	0.050	0.028
Men (n=102)	8.84	7.139	8.79	7.854	0.929	0.006	0.003
Women (n=48)	11.27	6.961	10.02	7.373	0.245	0.174	0.080

* St. Dev = Standard Deviation;

† P value;

‡ d = Cohen's D;

§ r = Effect size

Table 4 shows how the pain variable diminished at the first tube replacement, compared to that presented before implanting the nephrostomy tube. This difference is statistically significant (p <0.01) when analyzing the whole sample (1.83 ± 2.648 vs. 1.23 ± 1.781; Ttest = 2.707; p > 0.05). However, the size of the resulting effect was low (r = 0.13). Without reaching a statistically significant difference, women presented more pain than men (1.61 ± 2.599 / 1.23 ± 1.898, Ttest = 1.460, p = 0.147 in men pre/post; vs. 2.31 ± 2.714 / 1.25 ± 1.523; Ttest = 2.600, p > 0.05 in women pre/post).

**Table 4:** Pain pre and post-procedure. Santander, Spain, 2016-2018

	Pre-procedure		Post-procedure		Mean	St. Dev*	Mean
	Mean	St. Dev*	Mean	St. Dev*			
Total (n=150)	1.83	2.648	1.23	1.781	0.008	0.26	0.13
Men (n=102)	1.61	2.599	1.23	1.898	0.147	0.167	0.08
Women (n=48)	2.31	2.714	1.25	1.523	0.012	0.48	0.23

* St. Dev = Standard Deviation;

^†^P value;

^‡^d = Cohen's D;

^§^r = Effect size

Table 5 shows the mean values (before the nephrostomy tube implantation and at the first replacement) in the five areas through which the EuroQol-5D questionnaire assesses quality of life. The results indicate that there is higher post-implant mean values (and therefore worse perception of quality of life) in four of the five dimensions (mobility, personal care, daily activities and anxiety/depression). These results suggest a worsening of the quality of life of these variables in the first tube replacement, the results being statistically significant in all of them (p <0.05) with a large effect size (d >1), which indicates a correlation in all variables, except for mobility, with a moderate effect size (r = 0.40). It should also be noted that there is no correlation between the pain experienced by the patients before and after the implantation of the nephrostomy tube.

**Table 5:** Variables of EuroQol-5D questionnaire. Santander, Spain, 2016-2018

	Pre-procedure		Post-procedure		Mean difference pre/post^†^	P value^‡^	d^§^	r^||^
	Mean	St. Dev.*	Mean	St. Dev.*				
Mobility	1.11	0.339	1.28	0.493	-0.167	0.00	0.40	0.19
Personal care	1.09	0.355	1.68	0.616	-0.591	0.00	1.19	0.51
Daily activities	1.17	0.440	1.93	0.614	-0.760	0.00	1.42	0.57
Pain/ discomfort	1.43	0.628	1.43	0.584	0	1.00	0	0
Anxiety /depression	1.25	0.533	1.85	0.653	-0.593	0.00	1.00	0.44

* St. Dev = Standard Deviation;

† Mean difference pre/post = difference between mean values pre and post implantation of the tube (negative values indicate worse quality of life post-implantation);

‡ P value;

§ d = Cohen's D;

|| r = Size effect

## Discussion

The results of the present study show that patients living with a nephrostomy tube have reduced quality of life both globally and stratified by gender, confirming our first hypothesis, since the relationship between the placement of a nephrostomy tube and the decrease in the quality of life was statistically significant.

It should be noted that women had lower levels of quality of life, both before and after the procedure, similar results to those reported in other studies that evaluated quality of life according to gender^(^ 30 ^-^ 31 ^)^. Other sociodemographic variables, such as being single, divorced or widowed, lead to a worse perception of quality of life than married patients. Some of the reviewed studies^(^ 32 ^-^ 33 ^)^ support this theory. However, there are other similar studies^(^ 34 ^)^ that show data in which men and women have similar levels of quality of life while living with a particular pathology. Receiving adequate information about their process, emotional support, and the presence of a multidisciplinary team that not only meets the needs arising from their health episode but also the physical and psychological changes they may suffer, helps maintaining a good quality of life.

Living with nephrostomy tubes causes a negative impact on patients’ quality of life. Of the five areas assessed by EuroQol-5D, four of them are significantly diminished. Performing daily activities is the most altered dimension. Others, such as personal care and mobility, are affected to a greater or lesser extent. Regarding anxiety and pain that these patients present, the Beck Anxiety Inventory and the visual analog pain scale assessment at the first tube replacement show that both variables decreased in general compared to those presented before of the procedure. It is an unexpected and contradictory finding of the difference in pain outcome by analyzing it using the EuroQol-5D questionnaire, which shows no differences before and after, and the analogue visual scale of pain, which shows a clear reduction in pain when evaluated at the first tube replacement. Therefore, based on both results, the second hypothesis, that the level of anxiety and pain would increase at the first tube replacement, compared to the levels of pain and anxiety presented before implantation, was not confirmed. These result coincides with other studies^(^ 35 ^-^ 38 ^)^. Regarding the gender difference in anxiety, other articles^(^ 39 ^)^ suggest that there may be factors intrinsic to the female gender that predispose them to present more comorbidities, such as biochemical, hormonal and/or social aspects, that explain the differences per genus. Moreover, while feeling threatened by the disease, the historical role of the primary family caregiver, who has historically represented women, may be a cause or explanation for the increased anxiety levels, facing the likely reality that they will not be able to exercise this very role during the time they bear the tube^(^ 40 ^)^. However, the literature is not conclusive in this respect. This result, which reflects greater anxiety before the nephrostomy and its decrease in its first replacement, suggests that a possible health education or simply more information prior to the procedure could reduce the anxiety that the patients present in the moments before entering the intervention room, as described in other studies reviewed by the authors^(^ 41 ^)^. Prior consultation is the fundamental pillar where health education should begin, providing all the information, not only focused on the implantation of the tube, but in a holistic way, allowing the patient to express not only the doubts derived from the procedure he/she is being submitted, but also their yearnings, problems, etc. Some of the articles reviewed^(^ 42 ^-^ 43 ^)^ reflect the importance of reliable information before the procedure, which reduces anxiety levels before surgery. For this reason, we believe that a nursing consultation, where the patients receive the complete and necessary explanation, would reduce the levels of anxiety before the implantation of the tube and during all the time in which they live with the nephrostomy^(^ 44 ^)^.

The pain experienced by patients is one of the most important aspects before and after the procedure, with the result that, although the perceived quality of life is lower when the nephrostomy tube is used, the pain presented before decreases when the percutaneous intervention is fulfilled. It is important to note that, according to the results obtained, women present higher levels of pain than men, both before and during the time they live with the tube. Several studies show similar results that respond to this higher degree of pain. As with anxiety, psychobiological factors can explain this result. On the other hand, men may have higher pain thresholds than women, which could correspond to gender-related stereotypes that occur in cultures and/or societies where men repress certain emotions and actions, including pain, as endorsed by some studies^(^ 45 ^-^ 48 ^)^.

In addition to the negative impact of anxiety and pain presented by patients during the time they carry the tube, one of the most negatively affected areas is work. It should be noted that of all people who had worked before the nephrostomy tube was implanted, a high percentage of them are on medical leave, which shows not only the degree of physical impairment, but also the enormous social and labor impact.

The main strength of this study is to address the lack of research that specifically evaluates the quality of life, anxiety and pain in patients with nephrostomy tubes, verified by the extensive literature review performed by the authors. In addition, the assessment pre and post implantation of the tube was performed always by the same researcher and under the same conditions for all study participants, before undergoing nephrostomy (at the nursing visit) and at the first tube replacement (six weeks after the implantation), thus, guaranteeing the reliability of the outcomes. As limitations, we highlight the use of EuroQol-5D, a questionnaire that measures the quality of life in general. Perhaps, the use of more specific questionnaires, such as the Quality of Life Questionnaire for Patient with Ostomy (QOL-O), would have assessed the quality of life in a way that was more oriented to the problems that the target patients of this study could present. We believe that having modified the values ​​of the EuroQol-5D visual scale, which measures the patients’ quality of life, may be a limitation if we compare this study with others of a similar nature. In addition, although the University Hospital Marqués de Valdecilla is the reference center for the entire community of Cantabria (Spain), and where most nephrostomies are performed, another local hospital, Sierrallana, also performs some nephrostomy, but the authors of this article did not have access to this population. Another limitation of this study is that the influence of psychotropic drugs in improving anxiety at the first tube replacement was not analyzed in patients who used these drugs in comparison to those who did not. Such an analysis we hope to accomplish in future work.

## Conclusion

Living with nephrostomy tubes generates anxiety and has an important impact on patients’ quality of life. This is significantly reduced in all areas we evaluated (mobility, personal care, daily activities, pain and anxiety). It is necessary to devise strategies or interventions to minimize this impact, considering the different sociodemographic variables that reflect that there are certain populations more susceptible to a decrease in daily quality of life and have higher levels of anxiety. In addition, in view of the large number of people who are actively employed before the procedure, they are temporarily unable to perform their duties with consequent sick leave, this implies a high consumption of economic resources, not only direct health costs, but also non-health indirect costs due to lack of labor productivity. Our results are very useful to assess which aspects of daily life have been most altered and in designing a strategy of cognitive-behavioral intervention based on health education that can help to recover or, as much as possible, improve the quality of life of these people. Health education, both for the patient and directed to the main caregivers, can be a key pillar to support interventions that, if it is not possible to maintain the quality of life prior to nephrostomy, at least reduce to the maximum the impact that this procedure imposes.
